# Patterns of Lifetime Criminality in Mentally Disordered Offenders – Findings From a Nationally Representative Cohort

**DOI:** 10.3389/fpsyt.2021.564171

**Published:** 2021-07-29

**Authors:** Hedvig Krona, Henrik Anckarsäter, Thomas Nilsson, Björn Hofvander

**Affiliations:** ^1^Lund Clinical Research on Externalizing and Developmental Psychopathology, Child and Adolescent Psychiatry, Department of Clinical Sciences Lund, Lund University, Lund, Sweden; ^2^Department of Forensic Psychiatry and Center of Ethics, Law and Mental Health, Institute of Neuroscience and Physiology, University of Gothenburg, Lund, Sweden

**Keywords:** violent criminality, forensic psychiatry, lifetime criminality, criminal career, cluster analysis, MANOVA (Multivariate Analysis of Variance), MANCOVA, multivariate ANCOVA

## Abstract

**Background:** Treatment of mentally disordered offenders (MDOs) is challenging as their behavior and clinical conditions can be traced to a complex constellation of major mental disorders, substance use and antisocial lifestyle. Finding subgroups of these offenders, which could guide treatment and risk assessment, is desirable. There are few long-term, prospective studies of risk factors for persistent criminal behavior among MDOs.

**Aims:** The aims are (1) to provide a map of lifetime criminality in MDOs, (2) to identify subgroups of offenders, and (3), if such clusters exist, to test whether they differ in lifetime criminality and patterns of negative events during in-patient treatment.

**Methods:** Background data on all offenders from the Malmö University Hospital catchment area sentenced to forensic psychiatric in-patient treatment 1999–2005 (*n* = 125) was collected. Data on negative events during treatment (violence, threats, absconding and substance use) from date of admittance until discharge or until June 30, 2008 was gathered. Court decisions for 118 of the cohort-individuals were collected from the 1^st^ of January 1973 until December 31, 2013. We used hierarchical cluster analysis to identify subgroups and MANOVA-analysis to examine differences between these clusters on lifetime criminality variables and negative events. A MANCOVA was used to control for time in treatment.

**Results:** The cohort was sentenced to a total of 3,380 crimes (944 violent) during the study period. Median age at first crime was 20 years (range 15–72), and at first violent crime 27 years (range 15–72). A subgroup (*n* = 26) was characterized by childhood adversities, neurodevelopmental disorders and later substance use disorders and was more often associated with substance-related crimes, financial crimes and lower age at first crime. During treatment, this cluster showed higher rates of substance use and threats. When controlling for treatment time, no differences in negative events were found.

**Conclusions:** This study replicated findings from prison populations of the existence of a more criminally persistent phenotype characterized by early-onset neurodevelopmental and behavior disorders, childhood adversities and later substance use disorders. We did not find this cluster of variables to be related to negative events during inpatient treatment when controlling for length of stay.

## Introduction

Pathways to delinquent behavior, and for some, to a lifelong antisocial lifestyle have interested researchers for decades (1–3) as the economic consequences of crime are painstakingly high and as the suffering of the victims is immeasurable. Longitudinal, population-based studies have shown that an individual's propensity to commit crime varies through the lifetime, and that causes behind antisocial behavior are complex and multifaceted (4–6). A relatively small group of offenders are accountable for the vast majority of all crime convictions (7–9) and the risk factors for a long and intensive criminal career include male sex, childhood temperamental or self-regulation problems, adverse childhood experience, substance use disorders (SUD) and early-onset antisocial behavior (10–15).

To explore the emergence of criminal career patterns, the developmental taxonomic theory in its original outline (6) posits that two groups of offenders can be identified; a smaller group described as life course persistent offenders and a larger group of so-called adolescent-limited offenders. The former exhibits a high level of aggressive and antisocial behavior with an onset in childhood and persistence into adulthood, a skewed male-to-female sex ratio, a higher incidence of neurodevelopmental disorders (NDDs, including ADHD, autism spectrum disorders, tics disorder, learning disabilities, intellectual disability and conduct disorders), and childhood adversities. The latter, on the other hand, are thought to start their criminal careers during their teens by mimicking more antisocial peers and continue to do so up until young adulthood, when their criminal activities typically wane. Later research has shown that NDDs in themselves heighten the risk for the development of conduct disorder (4) and antisocial behavior (16–19), but also for major mental disorders and SUD (20, 21). Furthermore, conduct disorder is one of the strongest predisposing factors for SUD and all major mental disorders, including schizophrenia and bipolar disorder (21, 22) possibly due to shared genetic vulnerabilities (23) and early adversity (24).

The previously identified heightened risk of violent behavior associated with schizophrenia and other psychotic disorders have in later studies emerged as magnified by comorbid SUD (25–27), but also a history of conduct disorder (28). Absolute rates of violent crime over 5–10 years in individuals suffering from schizophrenia varies between 6 and 10 % and to more than 10 % in individuals with SUD (12, 27, 28). In a study of forensic male inpatients diagnosed with psychotic illnesses, only a minority of the patients showed aggression either at baseline (one in about 15) or during the period of time covered by the study (one in 43) (29). Similar numbers have been shown for psychotic patients and regular inpatient units (30). In studies of inmates, having a major mental disorder has been shown to be a risk-factor for reoffending (15), but so are childhood adversities (2, 31, 32), genetic factors, gene-environment interactions and epigenetic processes (33, 34). Thus, previous studies of persistent violent criminality have found multiple independent risk factors for reconvictions such as childhood adversities (17, 35, 36), SUDs (37) and major mental disorders (28, 38–40) which illustrates that various facets are operating in different pathways to promote violent behavior in sub-groups of offenders. Hodgins proposed (23, 41) that mentally disordered offenders (MDOs) may be posited according to one of three trajectories; (1) Type I offenders who exhibit an antisocial lifestyle from childhood years and onwards, prior to the onset of illness; (2) Type II offenders who, prior to the onset of the illness do not have an antisocial behavior yet develop one after illness ensues, and; (3) Type III offenders who suffer from a major mental disorder for several years until they commit a severe violent act. Type I is suggested to be more influenced by genes linked to both behavioral problems and major mental disorders, whereas types II and III are linked to neurological changes associated with the emergence of a major mental disorder, including effects of SUDs and medication (23). These findings have later been replicated (42) and the need for specialized treatment against both psychosis and aggression identified (43).

Prerequisites for a sentence to in-patient forensic psychiatric treatment in Sweden is the presence of a severe mental disorder calling for such treatment and that the crime was committed under its influence, and that the crime is severe enough to warrant a prison sentence. As a group, Swedish forensic psychiatric patients share many similarities. The majority are men of which about two thirds suffer from schizophrenia, almost all have had contact with psychiatric health services prior to their sentence and it is usually a violent crime that leads to forensic psychiatric treatment (44). In a previous study of the cohort (45), negative events (e.g., absconding [leaving without permission], violence, threats, and substance abuse) during in-treatment were described in relationship to length of stay. Other studies have suggested that homelessness and a previous conviction of assault may predict patient aggressive events (46), yet there is a lack of studies exploring both life course patterns of criminal behavior in MDOs as well as violent behavior in forensic psychiatric settings.

The aims of the present study are; (1) to map lifetime criminality in a total cohort of persons sentenced to forensic psychiatric treatment and to describe different criminal patterns, (2) to determine if forensic psychiatric patients constitute of clinically distinct groups of offenders based on lifetime clinical and background characteristics in this population, and (3), if such clusters exist, to test whether they differ in variations of lifetime criminality and patterns of negative events during inpatient treatment.

## Materials and Methods

### The UPPRÄTT-Malmö Study Cohort

The present study is part of the UPPRÄTT-Malmö project, which has followed a total cohort of all 125 individuals (101 men and 24 women) who were sentenced to forensic psychiatric in-patient treatment during 1999–2005. The group is nationally representative as it includes all consecutively sentenced individuals who at the time of the forensic psychiatric treatment belonged to the catchment area of the Skåne University Hospital, Malmö, which was demographically typical for all of Sweden at the time of the study. The cohort has been portrayed in greater detail in three previous papers; in a previous study by Andreasson et al. (45), the median length of treatment stay was shown to be 951 days (2.61 years) with negative events (for example absconding, violence, substance use) being described in 71 (60%) of all cohort individuals. The study further described the in- and out-patient phases of treatment with respect to negative events and known background factors. In a second study, the in-depth clinical characteristics were described (47), showing that almost one third of the cohort (*n* = 36, 29%) had a first-degree relative with a mental disorder of some kind and another third (*n* = 34, 32%) had been in contact with child- and adolescent psychiatric care when young. The paper further delved into risk prediction of relapse in criminality during a 10-year follow-up based on clinical and background data, where one finding was that patients with a restriction order was less likely to relapse into criminality. Lastly, Delfin et al. have described the incremental effects of neuroimaging data on risk prediction (48) based on data from the UPPRÄTT-cohort. The present study is the first to use data from a second wave of register-based follow-up.

All individuals in the cohort underwent either a Forensic Psychiatric Investigation (FPI) (*n* = 97, 78 %) or a Forensic Psychiatric Screening Report (FPSR) (*n* = 28, 22 %) prior to sentencing. Detailed descriptions of the Swedish criminal system and the forensic psychiatric treatment have previously been published (49, 50), but in summary, the Swedish Penal Code Chapter 30, § six states that a person who has committed a crime under the influence of a severe mental disorder shall at first hand be sentenced to another sanction than a prison sentence and that the recommendation is a sentence for compulsory forensic psychiatric treatment. The Swedish concept of a severe mental disorder is defined within a medico-legal discourse and overlaps with the clinical definition of a major mental disorder to some degree. A severe mental disorder is in most cases defined as various psychotic states yet with no discernment of the etiology of the psychosis, and all have in common symptoms such as a disturbed perception of reality, thought disturbances, confusion, delusions, and hallucinations. In some cases, the weight of the symptoms of other non-psychotic diagnoses in combination may be assessed as a severe mental disorder. In most cases, medication is avoided during the FPI in order to secure an accurate assessment. Seven individuals (6 %) were omitted from the analyses as data on lifetime criminality was missing and/or because they were deported from Sweden following the forensic psychiatric treatment. Thus, a total of 118 individuals (96 men and 22 women) aged 19–73 (median age 38), were eligible for inclusion into this study.

### Data Collection and Measures

Baseline data including background variables (e.g., SUD and psychiatric illness in first degree relative, migratory background, institutionalized before the age of 18, occupation, and housing), suicide attempts, and diagnostics of mental disorders including NDD, were gathered from the FPIs and FPSRs in accordance with a structured protocol. The variables were chosen as they either explicitly or indirectly were proxies of previously described risk factors of criminal behavior. At the time of the original forensic investigations, the Diagnostic and Statistical Manual of Mental Disorders 4th Edition (DSM-IV, (51) was in use. Thus, all psychiatric diagnoses were set with the semi-structured interviews (SCID I (52) by the forensic psychiatrist and SCID II (53) by the forensic psychologist) at the time of the forensic psychiatric investigation, according to the multiaxial system. In order to enable statistical analyses, sub-types of diagnoses are collapsed according to the overarching diagnostic categories of the DSM-IV. In the FPSRs, the personality diagnoses were set in clusters and therefore, statistical analyses do not portray specific personality diagnoses. Diagnoses set previous to the FPI were revised if deemed necessary. All individuals in the cohort were assessed to have at least one diagnosis severe enough to be included in the definition of a severe mental disorder, yet the majority (70 individuals, 59 %) had more than one diagnosis at the time of the FPI.

#### Criminality Data

Information on lifetime criminality was collected from the National Crime Register which is managed by the Swedish National Council for Crime Prevention. The registry contains information on all criminal convictions in Swedish lower courts since 1st of January 1973. The cohort's criminal history is thus known from this date up until the study's ending-point, 31st of December 2013.

All crimes were categorized as being either violent or non-violent. *Violent crimes* were defined as the following; murder and manslaughter, negligent homicide, assault, sex crimes, violation of a woman's integrity^1^, robbery, arson, extortion, kidnapping, illegal restraint, unlawful coercion, violence against an officer, unlawful threat against civilians as well as officers, obstructing the course of justice (in Swedish law defined as an act in which threat or violence is used to force a person to not participate in a trial), violation of knife and weapon legislations, violent resistance, riot and creating a danger to another. It also included all sex crimes such as rape of adult or child, sexual coercion, sexual exploitation of an individual in dependence, sexual molestation of adults and children and intercourse with an offspring. The definition also included attempted and aggravated forms of the aforementioned crimes.

*Non-violent crimes* were categorized based on the headings of the Swedish penal code as well as categories used by the National Council of Crime Prevention: theft and shoplifting, traffic violations (including driving under the influence), financial crimes (including fraud and counterfeit), drug- and alcohol-related crimes and minor offenses (most commonly damage to other people's property).

#### Data on Length of Stay and Negative Events During Forensic Psychiatric Treatment

Data on length of stay have previously been described in depth (45). By using a structured protocol, data on negative events that occurred during in-patient time was gathered. Negative events were defined as *absconding* (running away from staff or wards, or to not be compliant with conditions for permission to move freely about or leave the hospital area, not returning in time from a granted permission to leave the ward or the hospital area, or withdrawal of such permissions), *substance use* (both alcohol and drugs, detected by breath and urine analyses), *threats* (verbal abuse perceived as threatening by the recipient) and *violent behavior* (such as pushes, punches and kicks). The data includes threat and violent events with both staff and patients as recipients. An event was registered in the database if it had been affirmed in the hospital files.

In-patient treatment time was defined as time from when the court decision gained legal force until discharge or until 30th of June 2008, the end-point of the aforementioned study (45).

#### Clinical and Risk Assessments

To assess the risk of renewed criminal behavior and psychopathic personality traits, HCR-20 (54) and the Hare Psychopathy Checklist-Screening Versions (PCL:SV, (55)) were used clinically at the time of the inclusion in the study. The HCR-20 is a 20-item checklist used in structured clinical violence risk assessments, where items are rated on a three-point scale (“not present” to “definitely present”). In the current study, only the first 15 items (the historical and clinical items) were rated at the time of the FPIs as the last five risk management items would have required the set-up of an individual treatment and management plan, a procedure which was not done during the investigations.

The PCL:SV screens for psychopathic personality traits and is a 12-item rating scale which is highly correlated with the 20-item full version (56, 57). The items are scored according to the manual and rated on a three-point scale (0 = does not apply, 1 = may apply or in some respects applies, 2 = does apply) and the variables measure the interpersonal, emotional and behavioral aspects of the construct of psychopathy.

Only individuals who underwent a full FPI were assessed with the HCR-20 and PCL:SV. In most cases this was done by the FPI team but in 25 cases the assessments were made retrospectively by the research team based on the information gathered from the FPI files and from extensive file and register reviews in each case. Previous studies have shown that it is possible to reliable assess psychopathy (58) and risk factors (59) from file-based information.

### Statistical Analysis

All analyses were conducted using version 25.0 of the SPSS (60). Due to some missing background data, all presented percentage values are based on valid percentages.

#### Basic Descriptive Data

Analyses of dichotomous variables were done by χ^2^-tests and Fisher's exact test when any cell count was less than five. Mann-Whitney *U*-test was used for continuous variables as data was not normally distributed. All statistics were calculated using anonymized data, using two-tailed *p*-values. To measure effect size, Phi scores for χ^2^-tests and *r* for Mann Whitney *U* tests are presented. According to Cohen's model (61), an effect size of 0.20 is small, of 0.30 medium, and of 0.50 large when Phi is used, and when *r* is used 0.10 indicates a small effect, 0.30 a medium effect, and 0.50 a large effect.

#### Cluster Analysis

In order to explore whether the cohort could be divided into subgroups due to differences in lifetime clinical and background characteristics, a hierarchical cluster analysis was performed. This method was chosen as it allows the data to develop inherent associations, as the variables which are closest to each other will form clusters. The following dichotomous variables were entered in hierarchical cluster analyses as these variables corresponded to previous studies of developmental taxonomies and were considered fairly static and possible to affect the outcome variables of lifetime criminality; migratory background, sex, SUD in a first degree relative, psychiatric illness in a first degree relative, level of education, having been in contact with child- and adolescent psychiatry (CAP), being placed in social custody or a youth institution before the age of 18, and being diagnosed with a NDD, SUD, psychotic disorder or a personality disorder. Ward's method (62) was used to identify the relevant number of clusters. Measures of similarity between cases was calculated through squared Euclidian distances. One insignificant variable of the hierarchical cluster analysis was excluded at a time in a step-wise manner, starting with the one with highest *p*-value until all remaining variables had a *p* < 0.05. Due to missing data on various variables, 93 individuals were available for the cluster analysis and the subsequent comparisons between identified clusters.

#### MANOVA and MANCOVA

One-way between-groups multivariate analysis of variance (MANOVA) was chosen to investigate cluster differences in variables of lifetime criminality and in negative events during in-patient treatment. Preliminary assumption testing was conducted to check for normality, linearity, univariate and multivariate outliers, homogeneity of variance-covariance matrices, and multicollinearity. Since the variables exhibited a non-linear relationship, all continuous data were transformed in accordance with the natural logarithm and as such used in the subsequent analyses. Eventually, two MANOVAs were performed. The first one to investigate cluster differences in lifetime criminality variables grouped as violent crimes and non-violent crimes, where the latter were further sub-categorized according to the headings of the Swedish penal code into theft and shoplifting crimes, traffic-related crimes, financial crimes, drug- and alcohol related crimes, and minor offense, and finally, age at first crime registered at courts. The second one to investigate negative events during in-patient treatment in the forensic psychiatric hospital defined as number of substance use events, absconding, violent events, and threats. To further explore the data, three one-way multivariate analyses of covariance (MANCOVA) were performed. As extensions of the MANOVA of lifetime criminality, age at the time of the FPI and time from initiating in-patient treatment at the index-crime until end of study or death, were entered as co-variates in two separate MANCOVAs. By extending data on negative events during in-patient treatment, length of stay was used as the co-variate in the third MANCOVA. In all the inferential statistical procedures, a Bonferroni correction was made as a post-*hoc* analysis to determine what would be considered a statistically significant *p*-value. The continuous variables included in the MANOVA analyses were converted to z-scores in order to illustrate the distribution of the means of the variables in linear diagrams. Statistical analyses were performed using the SPSS 25.0.

### Ethical Considerations

The study was approved by the regional ethical review board in Lund (64/2007 and 2014/911). All data were anonymised using coded files, and the key code was kept separate from the study material and database. Since it is register-based and it would not be possible to contact most participants due to the length of time that has passed after finishing treatment informed consent was not considered necessary. The fact that contact could pose a risk to vulnerable subjects with mental health and/or legal problems was also considered in the ethical approval. When applying for the second ethical approval, the ethical board requested that the authors of this study announced the planned register study in mainstream media. The announcement went out in the two largest newspapers in the Malmö and Gothenburg area in the beginning of 2015.

## Results

### Data on Lifetime Criminality

The total number of crimes found in sentences committed by the cohort from the beginning of registries at the 1st of January 1973 until endpoint of study at December 31, 2013 was 3380 (median 16.5, range 1–185). Five individuals (4 %) had committed only one crime during the study period and for 16 individuals (14 %) the index crime was their first registered offense. The median age at first crime was 20 years (range 15–72 years). Of the non-violent crimes, thefts or shopliftings were most common followed by traffic offenses, drug- and alcohol related crimes, financial crimes and fraud, and lastly other minor crimes.

The total number of committed violent crimes in the cohort was 944 (median 5.5, range 0–47). The median age at first convicted violent crime was 27 years (range 15–72 years). Eight individuals (7%) had not been convicted of a violent crime. Eight individuals (7%) had been convicted of some form of lethal violent crimes (murder, manslaughter, negligent homicide and attempts thereof) and 14 individuals (12%) had committed arson. Ten individuals (8%) had committed in total 37 sexual crimes (median 1.5 crimes, range 1–21). Four of these individuals (3%) had committed sexual crimes against children.

### Cluster Analyses

**Figure 1:**
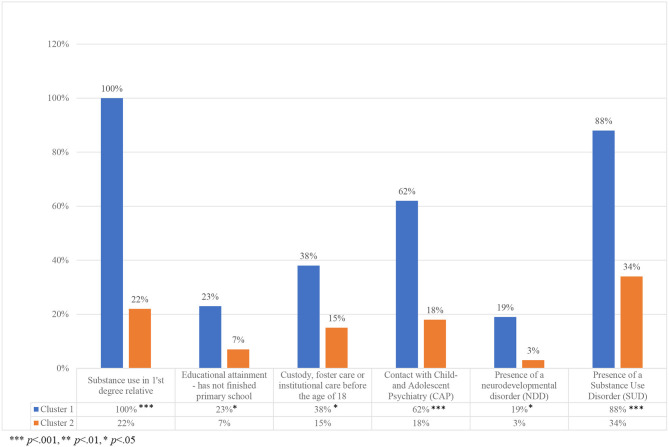
Variables in the cluster analyses.

**Figure 2:**
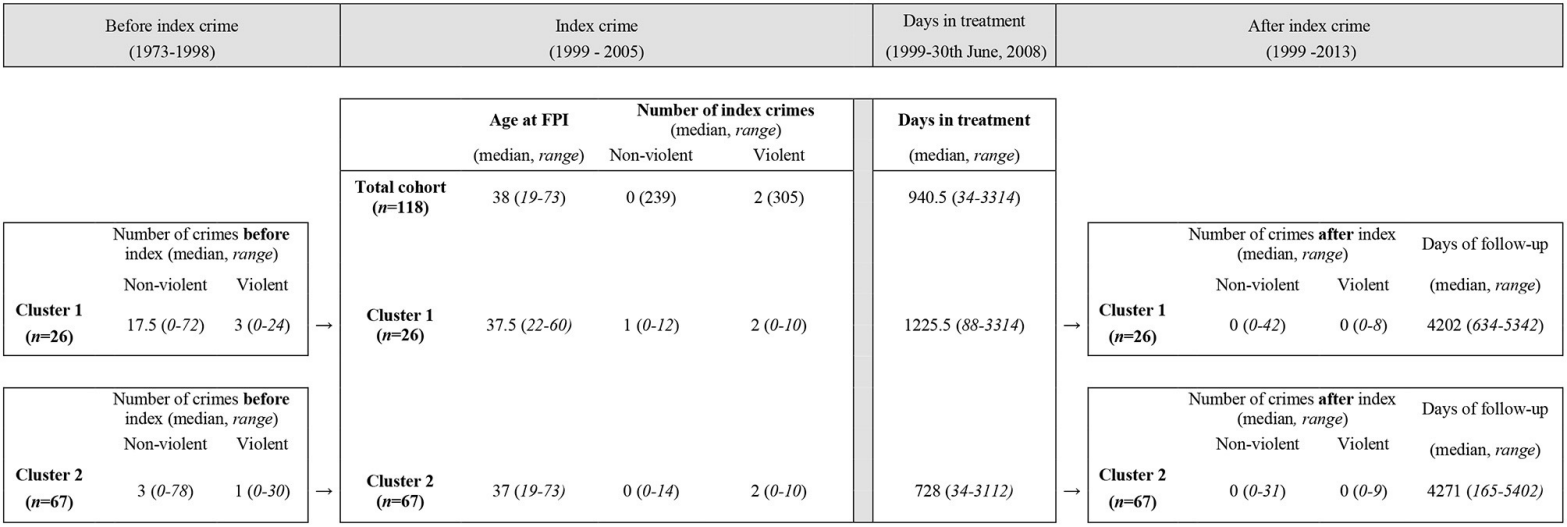
Lifetime criminality before, at, and after index crime.

Two clusters, 1 and 2, were identified based on background and clinical data, see Figures 1, 2. About a quarter of the cohort (*n* = 26, 28%) was grouped in cluster 1 and the rest, 67 individuals (72%), in cluster 2. Individuals in cluster 1 were more likely to have childhood onset problems or adversities, such as having a first degree relative with SUD, not having finished primary school, having contact with CAP, being placed in social custody or a youth institution before the age of 18, and having NDD. Presence of SUDs was also more prevalent among those in cluster 1.

In Table 1, the total cohort and the two clusters are described and compared on background and clinical variables not included in the cluster analysis. The first cluster consisted more often of individuals with a Swedish descent (*p* < 0.001), and they also had higher HCR-20 scores compared with cluster 2, both on total scores (H and C items combined, *p* < 0.01) and on historical scores (*p* < 0.01).

**Table 1:** Background and clinical characteristics of the cohort and comparisons between the two clusters.

	**Total Cohort**	**Cluster 1**	**Cluster 2**	**Phi**	***r***
**Variables**	***n* = 118**	***n* = 26, 22%**	***n* = 67, 57%**		
	***n* (%)**	***n* (%)**	***n* (%)**		
**Background characteristics:**					
Male sex	96 (81)	19 (73)^b^	57 (85)^b^	−0.139	
Migratory background	59 (50)	6 (23)^b^	41 (61)^b^^***^	0.342	
Occupation (work or studies) at the time of the FPI (*n* = 92)	6 (7)	1 (4)^a^	5 (8)^a^	0.068	
Housing (permanent or accommodation) at the time of the FPI (*n* = 92)	44 (48)	14 (54)^b^	30 (46)^b^	−0.076	
No of individuals who have made one or more suicide attempts at the time of FPI/FPSR (*n* = 93)	27 (29)	9 (35)^b^	18 (27)^b^	−0.077	
**Diagnosis according to DSM-IV** **(** **51** **)**					
Psychotic disorder	88 (75)	18 (69)^b^	49 (73)^b^	0.039	
Mood disorder	13 (11)	2 (8)^a^	11 (16)^a^	0.113	
Anxiety disorder or OCD	13 (11)	5 (19)^a^	7 (10)^a^	−0.118	
Personality disorder, any	33 (28)	11 (42)^b^	15 (22)^b^	−0.199	
Personality disorder, cluster A	6 (5)	4 (15)^a^	2 (3)^a^^*^	−0.227	
Personality disorder, cluster B	19 (16)	7 (27)^b^	7 (10)^b^^*^	−0.207	
Personality disorder, cluster C	1 (1)	0 (0)^a^	1 (2)^a^	0.065	
Personality disorder, NOS	9 (8)	2 (8)^a^	5 (8)^a^	−0.004	
**Risk Assessments**					
PCL:SV (55), (*n* = 89), Total score, Median (range)	11, (0–22)^c^	12, (0–21)^c^	10, (0–22)^c^		0.027
HCR-20 (54), (*n* = 88), H and C scores, Median (range)	19, (0–28)^c^	21, (0–28)^c^	16, (3–28)^c^^**^		0.122
HCR-20, Historical variables, (*n* = 88), Median (range)	12, (0–19)^c^	14, (0–18)^c^	11, (2–19)^c^^**^		0.132
HCR-20, Clinical variables, (*n* = 89), Median (range)	7, (0–10)^c^	7, (0–10)^c^	7, (1–10)^c^		0.022

*NOS, Not Otherwise Specified*.

*** *p < 0.001*,

** *p < 0.01*,

* *p < 0.05*.

a *Fisher's Exact test*.

b *Pearson Chi-square*.

c *Mann–Whitney U test*.

### Cluster Comparisons of Lifetime Criminality

The criminal careers differed between the two clusters that were identified. There was a statistically significant difference in a MANOVA between clusters 1 and 2 on the combined dependent variables covering criminality in a lifetime perspective, *F* (7, 85) = 2.46, *p* = 0.024; Wilks' Lambda = 0.832; partial eta squared = 0.168. When the results for the dependent variables were considered separately, two variables reached statistical significance, using a Bonferroni adjusted alpha level of 0.007; lifetime number of financial crimes, *F* (1, 91) = 12.03, *p* = 0.001, partial eta squared = 0.117, and number of alcohol or drug-related crimes, *F* (1, 91) = 8.85, *p* = 0.004, partial eta squared = 0.089. An inspection of the mean scores indicated that individuals of cluster 1 were more often convicted of financial crimes compared to those in cluster 2 (*mean (M)* = 3.42, *standard deviation* (*SD*) = 3.44 vs. *M* = 1.22, *SD* = 1.98), and of alcohol or drug-related crimes (*M* = 4.85, *SD* = 7.49 vs. *M* = 1.67, *SD* = 3.35).

To further test the validity of the identified clusters, two MANCOVAs were made. First, age at the time of the FPI was added as a co-variate. There was a statistically significant difference between the clusters on the combined dependent variables after controlling for age at the time of the FPI, *F* (7, 84) = 2.93, *p* = 0.009, partial eta squared 0.196, Wilks' Lambda = 0.804. When the results for the dependent variables were considered separately, only one variable reached statistical significance, using a Bonferroni adjusted alpha level of 0.007; age at first crime, *F* (1, 90) = 109.82, *p* = 0.000, partial eta squared = 0.550.

A second MANCOVA was made by entering time from initiating in-patient treatment at the index-crime until end of study or death, as a co-variate. There was no statistically significant difference between the clusters on the combined dependent variables, *F* (7, 84) = 0.701, *p* = 0.671, partial eta squared 0.055.

### Cluster Comparisons of Negative Events and In-Patient Treatment Time

A MANOVA was also performed to investigate cluster differences in negative-events during in-patient treatment at the forensic psychiatric hospital. In the analysis, four dependent variables were included: absconding, substance use, threats or violence during in-patient treatment. As in the first MANOVA, the independent variable was the two clusters.

There was a statistically significant difference between clusters 1 and 2 on the combined dependent variables, *F* (4, 88) = 2.57, *p* = 0.044, Wilks' Lambda = 0.896; partial eta squared = 0.104. When the results for the dependent variables were considered separately, the only difference to reach statistical significance using a Bonferroni adjusted alpha level of 0.0125, was number of events of substance use during in-treatment time, *F* (1, 91) = 7.36, *p* = 0.008, partial eta squared 0.075. Here individuals in cluster 1 were more often involved in using drugs or alcohol during in-patient treatment time (*M* = 4.31, *SD* = 5.07 vs. *M* = 1.93, *SD* = 4.50).

However, when length of stay was added as a co-variate in a MANCOVA, there was no statistically significant difference between the clusters on the combined dependent variables [*F* (4, 85) = 1.78, *p* = 0.106, partial eta squared 0.085].

## Discussion

This study of a nationally representative total cohort of MDOs sentenced to forensic psychiatric treatment had as a first aim to map lifetime criminality. The study individuals were markedly crime burdened as 96% had been sentenced for at least two crimes during their lifetime and as the median number of crimes during the lifetime was 16.5. Added to this, official crime registry data does not include the full extent of all committed crimes, only those that have led to a sentencing. It is therefore a fair assumption that there is a large quantity of criminal behavior not reported in this study and that the cohort as a whole could be described as having various forms of persistent criminal careers. Previous studies of life course patterns of criminal behavior have shown that a long criminal career is associated with low age at first committed crime (63), a finding that also applies to our cohort. This is consistent with theories of a heightened risk for criminal behavior during the life course by a progression of disruptive behavior through conduct disorder in adolescence to an adult antisocial lifestyle which does not subdue in adulthood (64, 65).

The subsequent aims of the study were to identify subgroups of offenders and to test if these clusters differed in patterns of negative events during in-patient treatment time and lifetime criminality. Through a hierarchical cluster analysis, we found a small, more crime-prone subgroup, characterized by substance abuse among their first-degree relatives, presence of NDDs, low educational attainment, previous contacts with CAP and out-of-home placements during childhood and adolescence. Later in life they also developed SUDs more often compared to the larger cluster. Previous studies have shown that genetic effects, prenatal risk factors such as *in-utero* exposure to alcohol and toxins and childhood adverse experiences such as familial psychopathology, maltreatment and neglect are potential risk factors for conduct disorder (2, 66, 67), which in turn is a risk factor for a criminal career later in life (68).

Interestingly, the two clusters did not differ in terms of different PCL:SV scores, which could have been expected. One possible explanation may be that the PCL:SV instrument was originally validated using non-psychiatric participants (57), and that the current cohort differs in demographic characteristics compared to them. Furthermore, previous studies of psychopathy in forensic psychiatric patients (69) have shown that PCL-scores tend to be in lower range, possibly related to factors such as medication and psychotic symptoms. In our small study group, low scores in general probably decreased our possibilities to detect differences between the two clusters, contrary to our expectations. As was described in the methods section, the R-variables of the HCR-20 were not rated as the instrument was not to be used as a clinical risk assessment tool. In order to reduce the risk of aggressive inpatient behavior, applying a strategy of making risk assessments on all individuals on inpatient psychiatric units and not just the actively aggressive ones, is recommended (70).

The study's cluster construction renders support to previous findings of associations between persistence in violence in offenders and SUD, low educational attainment and parental risk factors such as psychiatric disorders (7). The NDD-diagnoses were more prevalent in cluster 1, which corresponds to previous theories that neurodevelopmental aberrations are central in a more persistent group of offenders (6, 71). It is estimated that 5–10% in the general population has any type of NDD (72) but in violent offenders in prison, the prevalence of ADHD may be close to 50% and for autism spectrum disorders up to 10% (16). When testing the relationship of NDDs and the risk for violent criminality in a population-based register study, only ADHD and tic disorders were found to be risk factors (73), yet other studies have shown that both ADHD and autism spectrum disorders carry a risk of adverse outcomes such as behavioral disturbances, criminality and an antisocial lifestyle in adolescence and adulthood (17–19). The low number of diagnosed NDDs in this study is probably not a true representation of the incidence and considering the risk these diagnoses carry for continuous criminality, this study urges the importance of testing for these disorders.

When testing the clusters in MANOVA-analyses of lifetime criminality and number and type of negative events during in-patient treatment time, cluster 1 had a lower age at first crime, had committed a proportionately larger number of financial and drug-related crimes and also had more registered events of substance use during in-patient treatment time, compared to cluster 2 (all *p* < 0.05), though the latter statistical significance ceased when controlling for length of stay. A criminal lifestyle and SUD go hand in hand as crime may be a necessity in order to sustain a misuse of drugs and/or alcohol. Though we could not replicate three clusters exhibiting the criminal trajectories Hodgins proposed, the findings of the current study are in concordance with studies suggesting two subgroups of early and late starters (74–76) as the individuals of the more crime prone cluster 1 had an earlier age at first crime and a more extensive criminal career compared to cluster 2. This was further indicated when age at the time of the FPI was added as a co-variate to lifetime criminality as there continued to be a statistically significant adjusted mean difference between the two clusters. The importance of SUD found in the current study has also been proved in follow-up studies of Hodgins' typology (77) as SUD is in itself a known risk factor for criminal behavior (28). A recent study by Sariaslan et al. (78) found that the elevated risk of a psychotic individual committing a violent offense was found to be due to the same genetic influences that simultaneously elevates the risk to develop mental health problems, SUD and commit violent crime. This may suggest that the same set of genes contribute to both psychiatric symptoms and violent behaviors.

Using official conviction data always carries the risk of overlooking true crime rates. Studies comparing self-reports with official records have shown that offenders in reality have a lower age at first crime, longer criminal careers as well as a larger volume of committed crimes than what is found in registries (79). In addition to the plausibility of more extended criminal careers, a limitation in this current study is that registers of sentences passed before 1973 were not obtainable. This may give a false lower number of lifetime criminality as 24 individuals (20 %) were of legal age before 1973. As the number of individuals who were of legal age before 1973 were evenly distributed between the two clusters with no significant difference in any of the studied variables, and as the exclusion of these individuals would give a heavy impact on the possibilities to conduct statistical analyses, all individuals were included in the study.

In earlier studies (80), a history of previous criminality has been proven to be a potent predictor of both general crime as well as violent crime, yet these findings were only partially replicated in this current study as life history of violent crime did not differ significantly between the clusters. This may be due to the fact that almost all individuals were sentenced to forensic psychiatric in-patient treatment due to violent criminality. Added to this, the number of committed violent crimes differed greatly due to the heterogenous nature of the cohort. Another limitation of the study is the difficulty of measuring the potential effects prescribed treatment interventions and psychiatric medication might have had on violent behavior both previous to and during forensic psychiatric treatment. Research has shown that antipsychotic medication, especially such given in depot forms as well as mood stabilizers, reduce the risk of violent behavior (81, 82), particularly in individuals with both schizophrenia and conduct disorder (83). By offering MDO's interventions targeting both the mental disorders and the criminal behavior, forensic psychiatric caregivers may have reduced symptoms of distress and given the cohort individuals improved coping abilities and behavioral functioning (84).

In summary, the current study adds to a previous body of research by suggesting that there is a high risk, life course persistent phenotype not only in prison populations but also in forensic psychiatry. This subgroup is defined by an adverse childhood environment, early-onset antisocial behavior and psychiatric problems, poor school performance, out-of-home placements and later substance abuse. A larger and more sub-group focused study recruited to enhance group comparisons would have made comparisons clearer, yet the advantages of a consecutive, total cohort outweigh these limitations, as there are few such studies. The study adds important knowledge of the group of forensic psychiatric patients as a whole and illustrates the challenges the clinical teams have in assessing and treating them.

## Data Availability Statement

The raw data supporting the conclusions of this article will be made available by the authors, without undue reservation.

## Ethics Statement

The studies involving human participants were reviewed and approved by the study was approved by the regional ethical review board in Lund (64/2007 and 2014/911). Written informed consent for participation was not required for this study in accordance with the national legislation and the institutional requirements.

## Author Contributions

HK, BH, HA, and TN designed the study. HK collected the data and took the lead in writing the manuscript in close collaboration with and under supervision of BH. TN and BH supervised the statistical analyses made by HK. All authors provided critical feedback and helped shape the analysis, research and full manuscript.

## Conflict of Interest

The authors declare that the research was conducted in the absence of any commercial or financial relationships that could be construed as a potential conflict of interest.

## Publisher's Note

All claims expressed in this article are solely those of the authors and do not necessarily represent those of their affiliated organizations, or those of the publisher, the editors and the reviewers. Any product that may be evaluated in this article, or claim that may be made by its manufacturer, is not guaranteed or endorsed by the publisher.

## Footnotes

^1^Referring to the crime “*grov kvinnofridskränkning”*, which by encompassing several violent crimes (among others battery, molestations, threats and unlawful constraint) identifies the long-term harmfulness created by repeated acts of violence by a present or former spouse. The crime renders a more severe sanction than the sentences of each encompassed crime separately.
